# Incorporation of text recognition into trial it systems to safeguard against enrolment of ineligible patients or inappropriate reporting of adverse events

**DOI:** 10.1186/1745-6215-14-S1-O15

**Published:** 2013-11-29

**Authors:** Martin Dennis, Rustam Salman, Gillian Mead, Connor McGill, David Perry

**Affiliations:** 1 University of Edinburgh, Edinburgh, UK

## 

In the on-going multicentre FOCUS (http://www.focustrial.org.uk) and RESTART (http://www.RESTARTtrial.org) trials, researchers randomise patients and report adverse events via our bespoke web-based trial IT system.

Our protocols specify certain medical conditions, and concurrent medications, which make the patient ineligible e.g. epilepsy, anticoagulants. As well as asking the researchers to answer questions to confirm the presence or absence of particular criteria, we have also introduced text recognition as an added safeguard. For example, if when the researcher enters the list of patients' concurrent medications, they enter a drug which is incompatible with the IMP, then the text recognition system highlights this. Epilepsy is a contraindication in the FOCUS trial, so that if the researcher enters the name of any anticonvulsant drug, the system highlights the potential ineligibility.

Our protocols have identified certain adverse events (AEs) e.g. stroke, or heart attack, which may occur during follow-up and which should be reported as "outcomes" rather than via the AE reporting system. Also, to minimise the burden on researchers we have identified AEs which are common complications of the index disease or IMP, which don't need to be reported as an AE. If a researcher enters a term, for example "stroke" or "epilepsy", then our text recognition highlights this and the system recommends the more appropriate course of action. This aims to avoid inappropriate reporting of AEs with the attendant need for these to be reported to the sponsor and also followed up to resolution etc.

